# Case of an Encysted Tumor of the Eyelid, Which Was Found to Contain Hair

**Published:** 1785

**Authors:** 


*Ipfwich,*
Auguft 28, 1785.

---

VIII. *Cafe of an encyfted Tumor of the Eyelid, which was found to contain Hair. By Mr.* Goodwin.

A Young woman, aged twenty-five years, applied to me lately on account of a tumor, of the fize of a fmall walnut, on the upper eyelid, which, by its weight, clofed the eye, and of courfe obftructed her fight.

As it was moveable, and evidently of the encyfted kind, I recommended excifion. To this fhe readily confented, and accordingly I diffected out the tumor, which adhered anteriorly to the cellular membrane, and pofteriorly to the tunica conjunctiva. On examining its contents, they were found to confift of a thick, thick, dark, honey-like ſubſtance, intermixed with a conſiderable quantity of ſhort dark hair.

